# Ossifying fibromyxoid tumor of the hand: a case report and review of surgical intervention

**DOI:** 10.1093/jscr/rjag573

**Published:** 2026-07-13

**Authors:** Dev Patel, Diana Mastellone, Shuyue Ren, Gord Zhu, Ashley L Homan, Michael J Franco

**Affiliations:** Cooper Medical School of Rowan University, 401 South Broadway, Camden, NJ 08103, United States; Cooper Medical School of Rowan University, 401 South Broadway, Camden, NJ 08103, United States; Department of Pathology, Cooper University Hospital, 1 Cooper Plaza Camden, NJ 08103, United States; Department of Pathology, Cooper University Hospital, 1 Cooper Plaza Camden, NJ 08103, United States; Department of Plastic and Reconstructive Surgery, Cooper University Hospital, Camden, 1 Cooper Plaza Camden, NJ 08103, United States; Cooper Medical School of Rowan University, 401 South Broadway, Camden, NJ 08103, United States; Department of Plastic and Reconstructive Surgery, Cooper University Hospital, Camden, 1 Cooper Plaza Camden, NJ 08103, United States

**Keywords:** ossifying fibromyxoid tumor, OFMT, finger mass, S100

## Abstract

Ossifying fibromyxoid tumor is a rare mesenchymal neoplasm with variable malignant potential, most commonly arising in the subcutaneous tissues of the extremities and trunk. Involvement of the hand and fingers is exceedingly uncommon. We present a 59-year-old female with a 30-year history of a progressively enlarging mass of the small finger that became symptomatic due to neurovascular compression. Imaging demonstrated a partially calcified soft tissue mass without osseous involvement. The patient underwent surgical resection and pathology confirmed ossifying fibromyxoid tumor with diffuse S100 staining. The patient had an uncomplicated postoperative course. This case highlights the indolent yet progressive nature of these tumors, the challenges in preoperative diagnosis, and the surgical approach for excision.

## Introduction

Ossifying fibromyxoid tumor (OFMT) is a rare soft tissue and bone neoplasm of uncertain differentiation, first described by Enzinger *et al*. in 1989 [1]. These tumors most commonly arise in the subcutaneous tissues, although involvement of deeper soft tissues has also been reported [2]. Clinically, patients typically present with a slow growing, firm, well circumscribed mass [2].

Histologically, OFMTs are characterized by uniform round to spindle shaped cells arranged in cords or nests within a fibromyxoid stroma, often surrounded by a partial shell of mature lamellar bone [3]. They are classified as typical, atypical, or malignant based on nuclear grade, cellularity, and mitotic activity [4]. Radiologically they appear as well-defined, lobulated masses, with or without calcifications [3]. Although generally indolent, they can act unpredictably, with reported cases of local recurrence and, less commonly, distant metastasis [5]. Surgical excision is the primary treatment modality.

This report serves as, to our knowledge, the first documented instance of OFMT arising in the finger. This report highlights the diagnostic challenges and surgical considerations of OFMT of the finger.

## Case report

**Figure 1:**
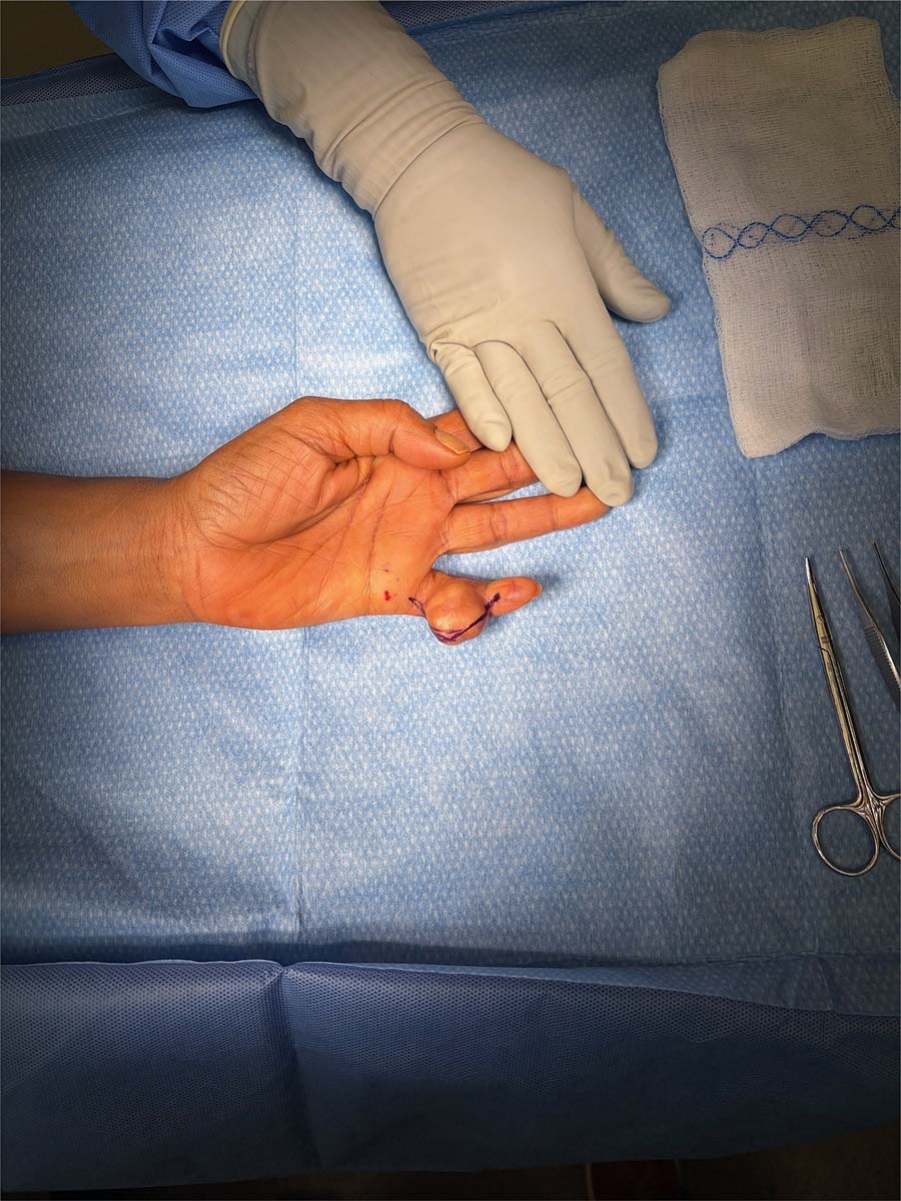
Intraoperative image demonstrating the gross presentation of the tumor.

A 59-year-old female with a past medical history of opioid use disorder and bipolar disorder presented to the hand surgery clinic for evaluation of a progressively painful mass in the fifth digit of her left hand. She reported that the mass had been present for ~30 years, but had recently increased in size and become painful. On physical examination, a 2 × 2 cm mobile mass was noted on the volar aspect of the fifth digit (Fig. 1). It was non-erythematous, non-pulsatile, and tender to palpation. The remainder of the physical examination was unremarkable.

**Figure 2:**
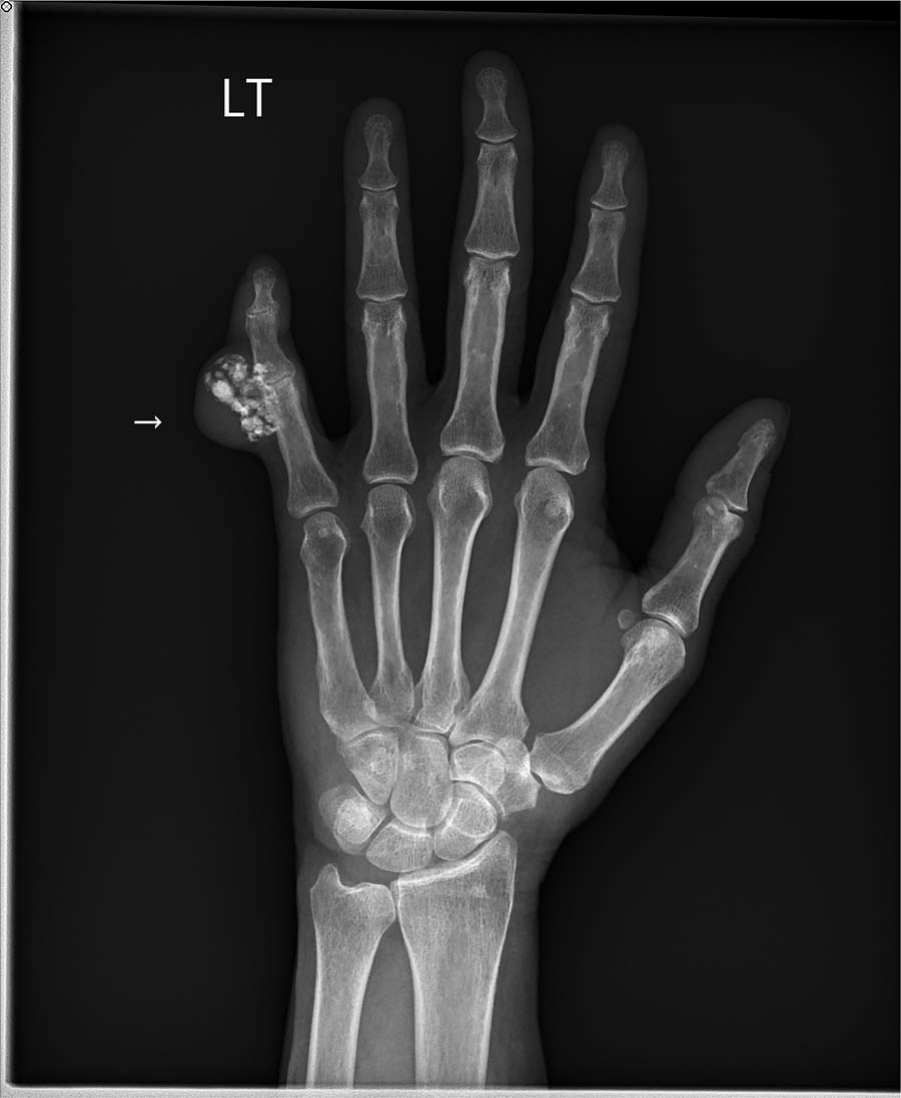
Plain radiographs demonstrated a 2 × 2 cm ovoid, partially calcified soft-tissue lesion.

Plain radiographs demonstrated a 2 × 2 cm ovoid, partially calcified soft-tissue lesion. The mass extended from the midportion of the proximal phalanx to the distal portion of the middle phalanx. No bony erosions or changes were noted (Fig. 2).

A discussion was held with the patient, and surgical management was jointly decided upon with the patient to obtain a definitive diagnosis and provide symptomatic relief.

### Surgical technique

**Figure 3:**
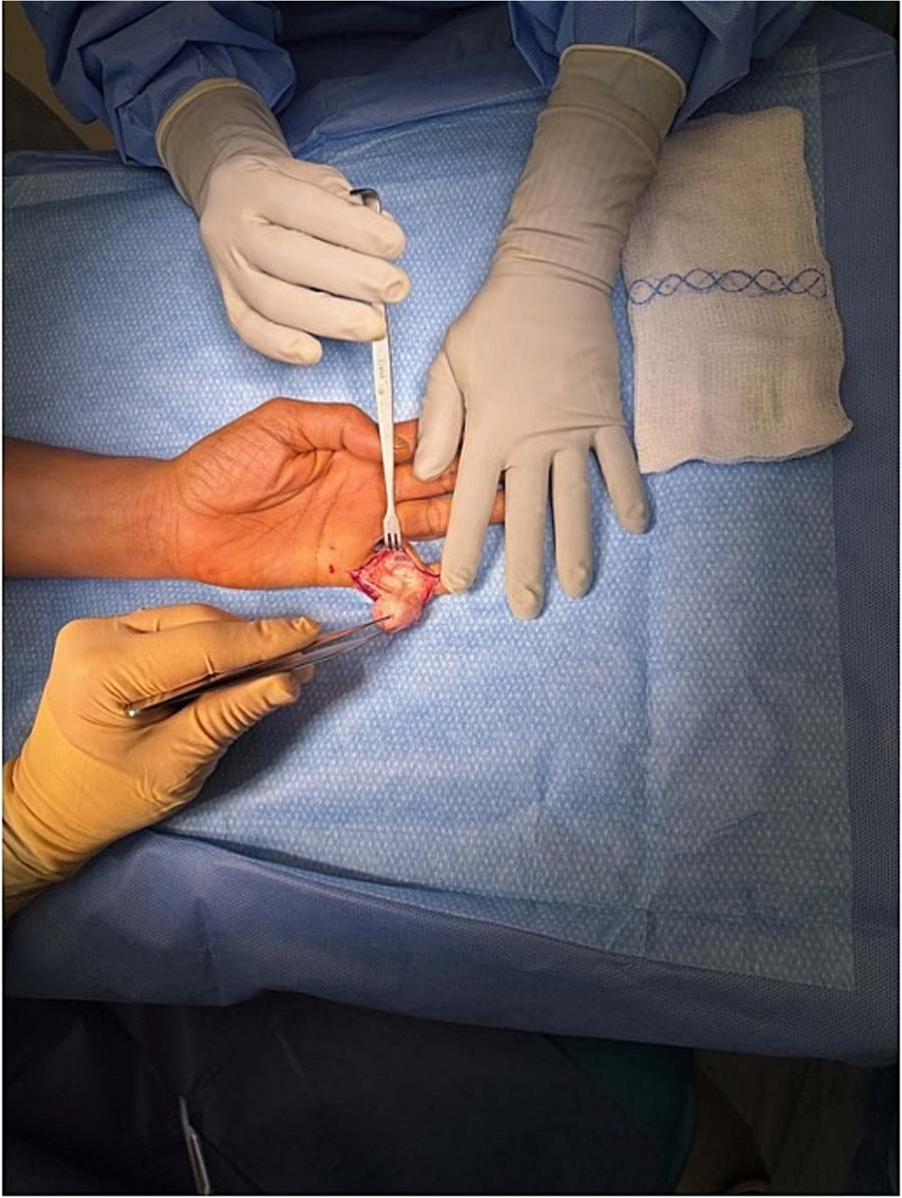
Intraoperative image demonstrating dissection of the tumor from underlying structures.

**Figure 4:**
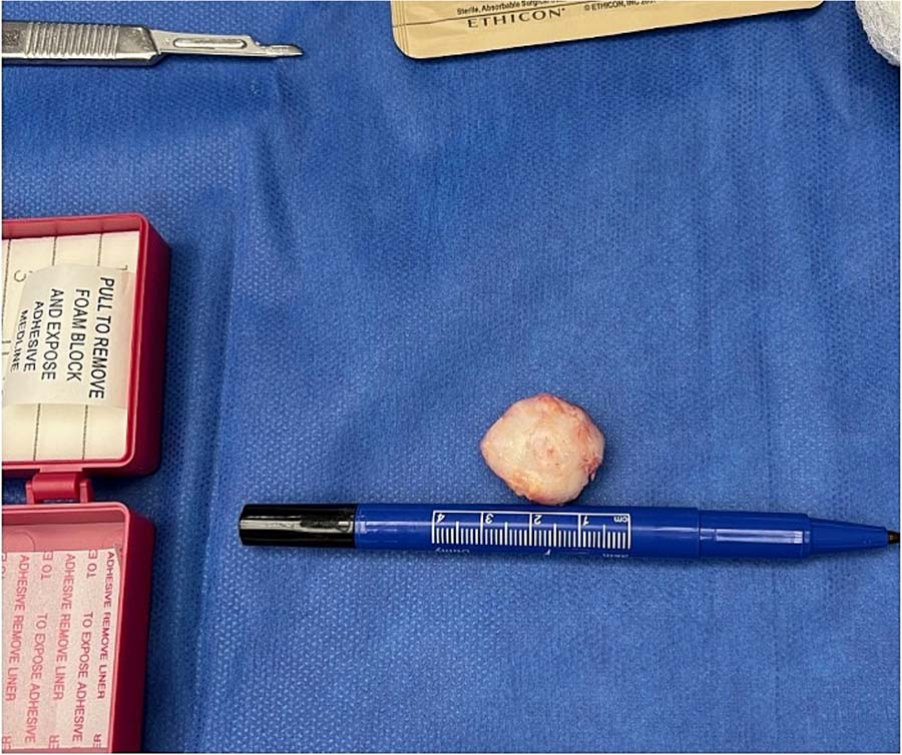
Intraoperative image showing the excised tumor measuring 2.5 cm.

The arm was exsanguinated using an Esmarch bandage, and a tourniquet was inflated to 250 mmHg. A zigzag incision was made over the mass, extending from the fingertip to the distal palmar crease. Dissection proceeded through the skin and subcutaneous tissue, with identification of the neurovascular bundles both proximally and distally. Soft-tissue flaps were carefully elevated over the mass; the overlying skin was notably thinned. The neurovascular bundle was attenuated and adherent to the mass. It was dissected free using 3.5× loupe magnification (Fig. 3). The mass was then circumferentially dissected and excised in a clean plane and sent for permanent pathology (Fig. 4). After tourniquet release, the wound was irrigated, and adequate digital perfusion was confirmed via capillary refill test. Thinned skin flaps were trimmed, and the wound was closed with absorbable sutures and dressed with Xeroform, gauze, Webril, and an Ace wrap.

She was seen for follow-up in the hand surgery clinic, where she reported complete resolution of her finger pain. She will continue with as needed follow-up appointments.

## Discussion

OFMT are a rare mesenchymal tumors that most commonly affect middle-aged male adults with a median age of presentation around 50 years [6, 7]. Patients typically present with a slow-growing, painless mass, often present for several years prior to diagnosis [6]. These tumors frequently arise in the subcutaneous tissues of the extremities, particularly the lower extremities, but have also been reported in the upper extremities, trunk, and head and neck [6, 7]. Tumor size is variable, typically ranging from 1 to 4 cm, although larger lesions have been described [6, 7].

Despite their generally indolent growth, OFMTs sometimes demonstrate unpredictable behavior. Local recurrence has been reported in up to 20%–25% of cases, often occurring many years after initial resection [6, 7]. Although uncommon, metastatic disease has also been described, particularly in atypical and malignant variants [7].

**Figure 5:**
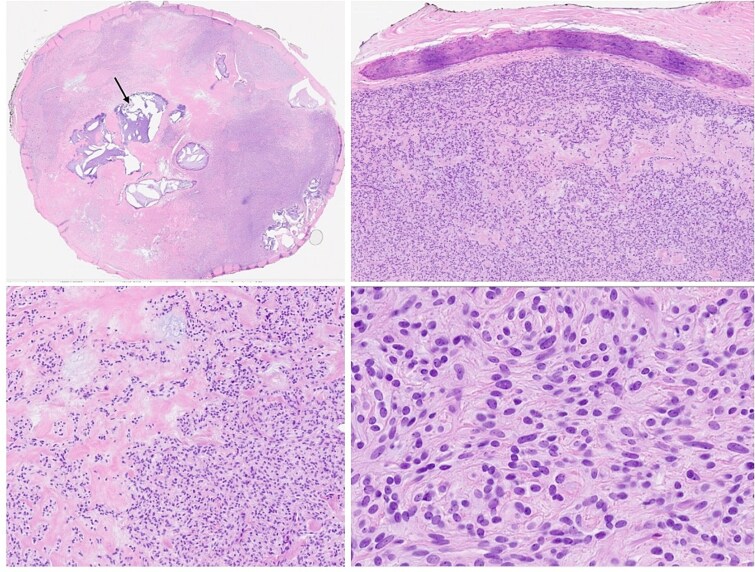
The left upper slide depicts a low-power view showing a well-circumscribed tumor with a surrounding capsule and a central area of degenerative ossification (arrow). The right upper slide depicts a shell of bone overlying the cellular neoplasm beneath it. The left lower slide is a medium-power view demonstrating the transition from a hypocellular, fibrotic region on the left to a hypercellular region on the right. The right lower slide is a high-power view showing tumor cells arranged in small clusters with relatively monomorphic oval to spindle-shaped nuclei and abundant eosinophilic to pale cytoplasm.

Histopathological evaluation in this case demonstrated a well-circumscribed tumor with a surrounding capsule and a central area of degenerative ossification (top left corner slide of Fig. 5). A peripheral shell of bone overlays the underlying cellular neoplasm (top right corner slide of Fig. 5). At intermediate magnification, there was a transition from a hypocellular, fibrotic region to a more hypercellular component (bottom left slide of Fig. 5). High-power examination revealed relatively monomorphic oval to spindle-shaped cells with abundant eosinophilic to pale cytoplasm, notably, no high-grade features, including increased mitotic activity, marked pleomorphism, or necrosis, were identified (bottom right slide of Fig. 5). These findings are consistent with a typical OFMT and correlate with the tumor’s indolent clinical behavior.

Molecular studies have characterized OFMT as a neoplasm due with recurrent genetic alterations [8]. Many tumors harbor the EP400-PHF1 gene rearrangements, which are present across typical, atypical, and malignant variants, and are thought to affect PRC2-mediated epigenetic regulation [9]. These rearrangements are also present in other mesenchymal tumors, supporting a shared pathogenic mechanism [9]. Immunohistochemically, S100 positivity, as was seen in this patient, has been associated with low-grade tumors [8]. Prior studies have found an inverse relationship between S100 expression and tumor grade, further supporting the benign course of this patient [6].

The prolonged 30-year history of slow growth, absence of high-grade histologic features, and favorable immunohistochemical profile suggest a low risk of recurrence or metastasis. Accordingly, follow-up was planned on an as-needed basis through shared-decision making with the patient.

Due to the rarity of OFMT, there are no standardized treatment guidelines or surveillance protocols. Surgical excision remains the mainstay of treatment. In cases of unresectable disease, positive margins, or aggressive features, systemic therapy may be considered. However, available data for choosing systemic therapy is limited, doxorubicin-based regimens have demonstrated poor efficacy, while a combination of gemcitabine and dacarbazine showed promising results in small series [10]. Further research is needed to better define optimal management strategies.
